# Estradiol induces bone osteolysis in triple–negative breast cancer via its membrane–associated receptor ERα36

**DOI:** 10.1093/jbmrpl/ziae041

**Published:** 2024-03-26

**Authors:** D Joshua Cohen, Cydney D Dennis, Jingyao Deng, Barbara D Boyan, Zvi Schwartz

**Affiliations:** Department of Biomedical Engineering, Virginia Commonwealth University, Richmond, VA 23284, United States; Department of Biomedical Engineering, Virginia Commonwealth University, Richmond, VA 23284, United States; Department of Biomedical Engineering, Virginia Commonwealth University, Richmond, VA 23284, United States; Department of Biomedical Engineering, Virginia Commonwealth University, Richmond, VA 23284, United States; Wallace H. Coulter Department of Biomedical Engineering, Georgia Institute of Technology, Atlanta, GA 30332, United States; Department of Biomedical Engineering, Virginia Commonwealth University, Richmond, VA 23284, United States; Department of Periodontics, The University of Texas Health Science Center at San Antonio, San Antonio, TX 78229United States

**Keywords:** tumor–induced bone disease, estrogen, SERMs, osteoclasts, osteolysis, cross-talk, bone resorption, ERα36, bone–tumor interface

## Abstract

Triple–negative breast cancer (TNBC) is thought to be an estradiol–independent, hormone therapy–resistant cancer because of lack of estrogen receptor alpha 66 (ERα66). We identified a membrane–bound splice variant, ERα36, in TNBC cells that responds to estrogen (E_2_) and may contribute to bone osteolysis. We demonstrated that the MDA-MB-231 TNBC cell line, which expresses ERα36 similarly to MCF7 cells, is responsive to E_2_, forming osteolytic tumors in vivo. MDA-MB-231 cells activate osteoclasts in a paracrine manner. Conditioned media (CM) from MDA-MB-231 cells treated with bovine serum albumin–bound E_2_ (E_2_-BSA) increased activation of human osteoclast precursor cells; this was blocked by addition of anti–ERα36 antibody to the MDA-MB-231 cultures. Osteoclast activation and bone resorption genes were elevated in RAW 264.7 murine macrophages following treatment with E_2_-BSA–stimulated MDA-MB-231 CM. E_2_ and E_2_-BSA increased phospholipase C (PLC) and protein kinase C (PKC) activity in MDA-MB-231 cells. To examine the role of ERα36 signaling in bone osteolysis in TNBC, we used our bone–cancer interface mouse model in female athymic homozygous Foxn1^nu^ mice. Mice with MDA-MB-231 tumors and treated with tamoxifen (TAM), E_2_, or TAM/E_2_ exhibited increased osteolysis, cortical bone breakdown, pathologic fracture, and tumor volume; the combined E_2_/TAM group also had reduced bone volume. These results suggest that E_2_ increased osteolytic lesions in TNBC through a membrane–mediated PLC/PKC pathway involving ERα36, which was enhanced by TAM, demonstrating the role of ERα36 and its membrane–associated signaling pathway in bone tumors. This work suggests that ERα36 may be a potential therapeutic target in patients with TNBC.

## Graphical Abstract

**Graphical Abstract:**
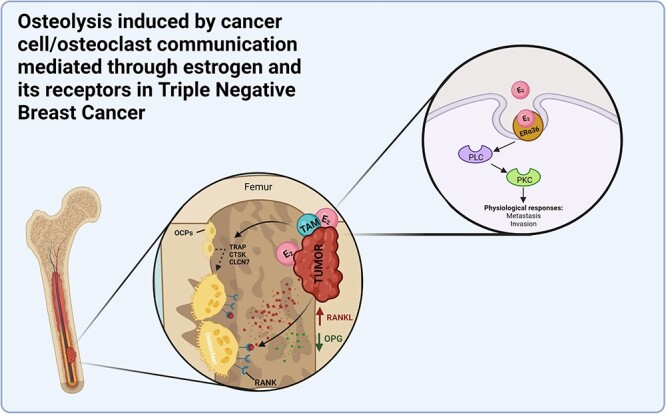

## Introduction

Breast cancer is an aggressive malignant type of cancer and the most common cause of cancer–related deaths in females. In 2022, the number of new invasive breast cancer cases was projected to be over 290 000, with more than 43 000 deaths. ^1^^,^^2^ Over 70% of these breast cancer diagnoses are estrogen receptor (ER) positive (ER+), with a 5–year survival rate of over 98% for localized tumors. However, once distant metastasis occurs, the 5–year survival rate decreases to <40%.^1^^,^^2^ Bone, the most common site of metastasis in breast cancer patients, is involved in ~70% of all metastatic patients,^1–4^ and patients with bone metastasis have significantly reduced survival rates of <15%.^1–6^

Patients diagnosed with triple–negative breast cancer (TNBC), accounting for ~12% of all breast cancer diagnoses, have an estimated 5–year survival rate of 24%.^2^^,^^7^^,^^8^ ER+ breast cancers are described as being less aggressive cancers as they are responsive to selective ER modulators (SERMs) like tamoxifen (TAM).^9–12^ TNBC is a subtype of breast cancer characterized by a lack of ER, specifically the canonical ER, estrogen receptor alpha 66 (ERα66), as well as the progesterone receptor and HER2. These cancers are described as aggressive because they not only evade apoptosis but they can be resistant to various treatments like TAM as well as radiotherapy and chemotherapeutic drugs.^10–13^

Cancer progression is a process driven by cancer cells’ ability to proliferate and evade apoptosis.^9–11^ Estrogen, particularly the predominant intracellular estrogen, 17β-estradiol (E_2_), can mediate cancer progression by inducing cell proliferation via binding to its intracellular receptors.^11–13^ The presence of ERα66 is most widely used as the molecular biomarker in evaluating diagnosis and prognosis as well as determining the course of treatment.^10–13^ While ERα66 negative (ER-) breast cancers do not express ERα66, they do express its splice variant ERα36 and sometimes ERα46. ERα36 contains ligand binding domains but does not include the transcriptionally active domains, AF-1 and AF-2.^10^^,^^14–17^

Previous studies in our lab demonstrated membrane–associated E_2_ signaling to stimulate the production of factors associated with metastasis by both ER+ and ER- breast cancer cell lines and to block apoptotic effects of anti–tumor agents.^10^^,^^18–20^ These studies used E_2_ conjugated to bovine serum albumin (E_2_-BSA), which is membrane impermeable, on the ERα66+ MCF7 breast cancer cell line and the ERα66-/ERβ- HCC38 breast cancer cell line.^21^ Both MCF7 and HCC38 cells exhibited a dose–dependent increase in protein kinase C (PKC) activity within 9 min of treatment with E_2_ or E_2_-BSA. This effect was specific to the PKCα isoform, not PKCβ, PKCδ, PKCε, or PKCζ, similar to the effect of E_2_-BSA on rat growth plate chondrocytes.^21^ Antibodies against ERα66 or ERβ did not block the E_2_–induced increase in PKC, suggesting another receptor might be involved. Later studies determined that this effect was mediated by ERα36 and resulted in increased proliferation, inhibition of apoptosis, and increased production of metastatic markers via rapid membrane–associated signaling pathways.^10^^,^^17^^,^^19^^,^^22^^,^^23^ ERα36, which is present in caveolae, acts via a phospholipase C (PLC)–dependent mechanism to activate PKC and downstream kinases.^10^^,^^17^^,^^19–21^^,^^23–29^

ERα36 plays a role in bone metabolism in postmenopausal women, resulting in increased proliferation and differentiation of osteoblasts and increased apoptosis of osteoclasts.^30^ Other studies have shown that ERα36 expression is associated with the acquisition of TAM resistance. Patients with hormone–responsive or ER+ tumors are most commonly prescribed TAM, an SERM.^15^^,^^18^^,^^19^^,^^22^^,^^31–34^ It acts as an ER antagonist, binding to ERs to competitively inhibit the estrogen binding site and blocking cell proliferation.^32^^,^^33^^,^^35^ TAM resistance occurs in ~50% of women and can be de novo or acquired over time.^32^^,^^36^^,^^37^

It is evident that the relationships between TAM, ERα36, and metastasis are complex. We have shown that TAM can inhibit PKC activity in human breast cancer cells,^21^ suggesting that it may be through its action on ERα36. Other studies showed that TAM could activate ERα36, resulting in increased expression of aldehyde dehydrogenase 1A1, a protein responsible for increasing the metastatic capabilities of breast cancer cells.^15^^,^^22^^,^^31^ Previous studies found that increased expression of ERα36 resulted in poor disease–free survival and disease–specific survival in two groups: patients that had ER+ breast cancers and were given TAM and patients that had ER- breast cancers regardless of whether they were given TAM or not.^31^ The results indicated that the presence of ERα36 resulted in TAM resistance and poorer prognosis.^31^^,^^33^ These findings suggest that TAM resistance may be caused by the drug’s inability to block estrogen signaling pathways mediated by ERα36.^31^^,^^34^ The mechanisms involved are unclear, although they may involve modification of the ERα66 binding mechanism, interference between ERα66 and ERα36 pathways, or altered cofactor and corepressor binding.^31^^,^^37^ The aim of using TAM in this study was to investigate the effects of estradiol in a model that mimics patients who are subjected to hormone therapy like TAM and experience an influx of estrogen from menstruation.

Bone metastasis involves a shift in the balance between bone formation and resorption, which is normally maintained through the homeostatic interactions of osteoblasts and osteoclasts. At the site of metastasis, osteolytic lesions in the bone shift the balance such that the formation of new bone is overwhelmed by bone resorption, leading to pain, fractures, and hypercalcemia. To successfully metastasize and invade bone, tumor cells must interact with osteoblasts and osteoclasts to create an optimal microenvironment for the growth of secondary tumors. However, this process has yet to be fully understood.

The goal of this study was to assess the role of ERα36 in mediating the effects of E_2_ in TNBC bone osteolysis. We first examined whether TNBC bone destruction is sensitive to E_2_. We adapted a previously established model of bone osteolysis developed in our lab^33^ for use in ovariectomized mice as the model system, thereby limiting the contribution of endogenous estrogen and mimicking the estrogen levels of postmenopausal women. We next evaluated the mechanism responsible for the increase in cancer cell–induced osteolysis by evaluating the effect of E_2_ on TNBC cell–mediated osteoclastogenesis and its potential signaling pathways. Finally, we evaluated the clinical relevance of TAM with or without estrogen treatment in TNBC bone osteolysis.

## Materials and methods

### Cell culture

The human breast cancer cell line, MCF7 and human TNBC MDA-MB-231 cells were obtained from the American Type Culture Collection (ATCC, Manassas, VA). Cells were plated on tissue culture polystyrene at a seeding density of 10 000 cells/cm^2^. MCF7 cells were cultured in Eagle’s Minimum Essential Medium (GIBCO, Thermo Fisher Scientific) lacking phenol red and supplemented with 10% heat–inactivated fetal bovine serum (FBS), 1 mM sodium pyruvate, 1% nonessential amino acids, and 0.1% insulin from bovine pancreas, 100 U/mL penicillin/streptomycin. MDA-MB-231 cells were cultured in RPMI1640 media lacking phenol red (Thermo Fisher Scientific) supplemented with 10% heat–inactivated FBS, 1 mM sodium pyruvate, and 1% penicillin/streptomycin. Human osteoclast precursors cells (OCPs) were obtained from Lonza (Basel, Switzerland) and cultured in OCP osteoclast precursor basal medium (Lonza) supplemented with 10% heat–inactivated FBS, 2 mM L-glutamine, 1% penicillin/streptomycin, 33 ng/mL M-CSF, and 66 ng/mL soluble RANK ligand (OCP Growth Medium SingleQuots Kit, Lonza). The murine macrophage RAW264.7 cell line was obtained from ATCC and cultured in Dulbecco’s Modified Eagle’s Medium containing 4.5 g/L glucose (GIBCO) supplemented with 10% FBS and 1% penicillin/streptomycin. All cells were cultured at 37°C and 100% humidity.

### ER characterization

Western blot analysis was used to characterize ER protein levels in MCF7 and MDA-MB-231 cells. Cells were plated at 10 000 cells/cm^2^ in a six–well plate and cultured to confluence. All cells were washed twice with 1x PBS and lysed in 200 μL radioimmunoprecipitation assay buffer (and resolved on 4%-20% Tris-glycine extended gels using gel electrophoresis. Proteins from gels were transferred onto a PVDF membrane (Bio-Rad, Hercules, CA) using the Bio-Rad Mini Trans-blot Electrophoretic Transfer Cell. Membranes were blocked and probed with a polyclonal ERα antibody (Thermo Fisher Cat# PA1-309), a polyclonal ERβ antibody (Abcam Cat# ab3576, Cambridge, United Kingdom), a polyclonal G–protein–coupled receptor 2 antibody (GPR30) (Abcam Cat# ab39742), and a glyceraldehyde-3-phosphate dehydrogenase (GAPDH) monoclonal primary antibody from Millipore-Sigma (Cat#MAB374, St. Louis, MO), as well as Li-Cor IRDye secondary antibodies from LI-COR Biosciences (Lincoln, NE). Membranes were then imaged using the Odyssey CLx Infrared Imaging System from LI-COR Biosciences. Schematics demonstrating the location of the exons encoding ER alpha, ER beta, and GPR30 target proteins are shown in Supplementary Figures S1 and S2.

To characterize the expression of ERs in MCF7 and MDA-MB-231 cells, wild–type cells were cultured on 24–well plates and cultured to confluence at which point they were harvested with TriZol (Invitrogen, Waltham, MA). RNA was extracted and quantified (Take3 Microvolume Plate, Biotek, Winooski, VT) and then used to synthesize cDNA libraries (High-Capacity Reverse Transcription Kit, Applied Biosystems, Waltham, MA). Quality of RNA was measured by the 260/280 ratio and was deemed pure with a ratio of 2. Gene expression was measured with real–time quantitative PCR using *Power* SybrGreen Master Mix (Applied Biosystems), at 40 cycles and gene–specific primers from Thermo Fisher Scientific (Supplementary Table S1). The optimal temperature for each gene of interest was determined by the amplification curves of a positive and negative control (RNAse free water) at temperatures from 55°C to 65°C. The temperature in which a single peak was observed only in the positive control and not the negative was deemed satisfactory. Expression levels were normalized to levels of GAPDH. A graphical representation of the ER alpha primer targets is shown in Supplementary Figure S1.

### Animal model

Female, 6-8–week–old athymic homozygous Foxn1^nu^ mice were obtained from The Jackson Laboratory (Bar Harbor, ME). The use of human cell implantation in an animal model requires an immunodeficient mouse model to prevent immune rejection. All animal procedures were approved by the Institutional Animal Care and Use Committee of Virginia Commonwealth University (Richmond, VA). Animals were fed a phytoestrogen free diet (Harlan Laboratory, Indianapolis, IN). A priori power analysis was conducted using the G*Power program. In order to determine significance of *a* = .05 and a power of 0.8, the minimum sample size required for the effect size was *n* = 8 for a one–way ANOVA.^38^

### Effect of estrogen on TNBC–induced osteolysis in ovariectomized mice

A total of 24 female 8–week–old mice were used for the experiment. Mice were randomly assigned to one of three groups (*n* = 8/group): sham, ovariectomy, and ovariectomy treated with E_2_. Sixteen mice were surgically ovariectomized and eight mice received a sham ovariectomy by the Jackson Laboratory at least 1 week before shipping them to our laboratory. Once received, mice were acclimatized in the vivarium for a minimum of 4 days. One mouse in the sham group did not survive and was therefore removed from the study.

There are various models of bone metastasis in vivo described in the literature that focus on injected delivery of tumor cells directly into the marrow cavity of the tibia.^39^^,^^40^ However, when we conducted a preliminary study using direct injection, we found that the method did not yield adequate tumor formation. We modified tumor cell delivery to improve the tumor engraftment within the bone, in which a small region in the femoral marrow cavity is ablated and the cells are implanted into this space.^33^ This design was used in the present study.

All mice underwent a distal femur marrow ablation procedure followed by inoculation of tumor cells into the medullary canal. Mice were anesthetized in a chamber of 5% isoflurane in 400 mL oxygen and maintained using 2% isoflurane with nosecone in 400 mL oxygen. Sustained–release Buprenorphine was administered via subcutaneous injection. Surgical inoculation of tumor cells into the bone marrow was performed as previously described.^33^ A medial incision to the patella was made to localize the distal femur. The periosteum was elevated, and a hole was drilled into the diaphysis of the distal femur using a number 4 dental burr attached to a low–speed drill in order to gain access to the marrow cavity. The marrow was flushed out from the distal canal by repeated irrigation with sterile saline. A suspension of 2 million MDA-MB-231 cells in isotonic saline was injected into the medullary defect and then seal with bone wax (Ethicon, Raritan, NJ). This bone marrow implantation closely mimics clinical situations observed with bone metastasis at later stages, thereby only evaluating osteolysis as an indicator of bone invasion.^41^^,^^42^ The periosteum and overlying muscles were replaced and the skin was re-approximated and closed with 7 mm wound clips. The E_2_–treated ovariectomy group mice were given subcutaneous implantations of 0.72 mg (0.012 mg daily) osmotic pumps (Innovative Research of America, Sarasota, FL) with a 60–day release of 17β-estradiol. After 8 weeks, all mice were euthanized and legs were harvested for μCT and histological analysis.

When developing this model,^33^ osteolysis was only observed in the final 3 weeks of the study. However, extracortical tumor growth became apparent during the 8th week of the study. A postsurgical time course longer than 8 weeks was not possible because of the severity of the fractured bones and our predetermined humane endpoints observed in a preliminary study. Prior to 8 weeks of growth, the differences between groups were limited, requiring an increase in the number of animals used. Therefore, to lessen the number of animals required, the 8–week end point was used. We considered performing a linear assessment of osteolysis using in vivo microCT. However, because repeated exposure to radiation had potential to be a confounding variable by affecting tumor growth,^43^ the decision was made to evaluate the final tumor volume in the final 8th week of the study.

### MicroCT analysis

Femurs were isolated and fixed in 10% neutral buffered formalin before scanning using the Bruker Skyscan 1173 MicroCT system and Skyscan Control Software. Images were scanned at a resolution of 2240 × 2240 pixels (with each pixel being 14.74 μm in size) and an energy level of 105 kV. The scans were reconstructed using NRecon Software version 1.6.9.17 and viewed using CTAn software version 1.14.4.1. 2D and 3D renderings of the tumor were then created using CTVox software via manual identification of the tumor edge on each cross–sectional image. Each of the samples was categorized by two independent non–blinded scorers as having osteolytic lesions, tumor, and/or pathologic fracture and averaged between the two scores. Bone volume was quantified as a fraction of treatment leg volume versus control leg volume. Briefly, the scans were binarized as black and white images, and excess noise was removed before data were quantified in a text table. Tumor volume was quantified by manually outlining the regions of interest over the entirety of the tumor as indicated by bone–free space. The volume was then quantified by the summation of pixels within the tomographic planes. The area of interest was identified by isolating the top of the femoral head and inactivating the next 300 μm cross–sectional slices from the analysis to allow for data homogeneity. The remaining slices were used for data analysis. MicroCT is not a precise measurement of tumor volume, but when comparing groups using the same evaluation method, microCT allows detection of smaller changes between groups than are possible using caliper measurements. MicroCT combined with histological analysis are common measurements of tumor volume. Other measurement methods, including in vivo luminescence and fluorescent–based tracking, would be beneficial in determining the location and number of cells within the tissue. However, to assess tumor volume, osteolysis, fracture, and breakdown of the cortical bone, microCT was ultimately chosen.

### Histology

Femurs were fixed in 10% neutral–buffered formalin and then decalcified using Decal bone decalcifier (StatLab, McKinny, TX) for 24 h. Samples were then rinsed thoroughly and dehydrated through a series of 95% and 100% ethanol and xylene washes and embedded in paraffin blocks, and cut into 5 μm sections. Sections were then placed on histobond slides (VWR, Radnor, PA), deparaffinized, rehydrated, and stained with hematoxylin (VWR) and eosin-Y (Thermo Fisher Scientific). Coverslips were mounted with toluene–based mounting media and dried flat. Images were taken using Zen 2012 Blue Edition. Qualitative analysis of histological slices was used to confirm osteolysis and pathological fracture.

### Effect of estrogen on cancer cell–induced osteoclastogenesis

To examine the effect of E_2_ on TNBC–induced osteoclastogenesis, two sets of experiments were performed. We first determined if the conditioned media (CM) from E_2_-BSA–stimulated TNBC cells could increase osteoclast activity by measuring degradation of collagen. In order to focus on ERα36 and membrane–associated signaling pathways, MDA-MB-231 cells (ERα66 negative; ERα36 positive) were cultured in 24–well plates to confluence and treated with vehicle, 10^-7^ M E_2_-BSA, or 10^-8^ M E_2_-BSA (Sigma Aldrich, St. Louis, MO). Previous studies showed that estradiol uses rapid membrane–mediated signaling actions via ERα36 to regulate downstream biological effects.^10^^,^^17^^,^^19–21^^,^^23^^,^^28^^,^^33^ In vitro, these signaling pathways were activated within 9 min of treatment and observed in normal cells as well as cancer cells.^20^^,^^26^^,^^29^ Therefore, all in vitro studies used a 9–min estradiol treatment. E_2_-BSA is unable to enter the cell.^16^^,^^21^^,^^28^^,^^44^ However, up to 5% of the E_2_ in E_2_-BSA is unconjugated and thereby able to enter the cell to bind to nuclear receptors.^29^^,^^45^ The 9–min exposure to E_2_-BSA limited this potential for artifact. In addition, cells were also co-treated with or without 1 μg/mL anti–ERα36 antibody (Alpha Diagnostic Cat# ERA361-A, Burlington, NC) to block the effects mediated by this ER variant. Following the 9–min exposure, the media from MDA-MB-231 cells were replaced with fresh media.

In parallel, human OCPs were seeded onto the collagen–precoated OsteoLyse Plates (OsteoLyse Assay Kit, Lonza) at a seeding density of 10 000 cells/cm^2^. Twenty–four hours after MDA-MB-231 cells were treated with vehicle, 10^-8^ M, or 10^-7^ M E_2_-BSA ± anti–ERα36 antibody, the replaced CM were collected and added to OCPs. After 7 days, the release of europium–labeled collagen into the supernatant was measured via time resolved fluorescence according to the OsteoLyse Assay Kit protocol. This method of evaluating osteoclast activity examines the resorption the matrix observed during the resorption process.

The second set of experiments evaluated which aspects of osteoclastogenesis were affected by the TNBC cells. For this study, osteoclasts were differentiated from RAW264.7 murine macrophage cells (ATCC) under 100 ng/mL receptor activator of nuclear factor kappa–B ligand (RANKL) (Biolegend, San Diego, CA) treatment for 48 h. MDA-MB-231 cells were cultured in 24–well plates to confluence and treated with vehicle or 10^-7^ M E_2_-BSA (Sigma Aldrich) for 9 min at which point media were replaced with fresh media for 24 h. CM from the MDA-MB-231 cells were collected and mixed 1:1 with RAW264.7 cell growth media. The RAW264.7 cells were incubated for either 24 or 48 h before being harvested with TriZol (Invitrogen). Gene expression for various osteoclast activation and differentiation markers was quantified through real–time quantitative PCR as described above with gene–specific primers (Supplementary Table S1). A schematic of the primer target locations is shown in Supplementary Figure S3.

### Effect of estrogen on osteoprotegerin, interleukin-6, and RANKL production

To further examine how TNBC cells modulate osteoclastogenesis, different secreted proteins known to play a role in osteoclastogenesis were measured. Confluent cultures of MDA-MB-231 cells were plated on 24–well plates, treated with vehicle, 10^-8^ M, or 10^-7^ M E_2_-BSA for 9 min, and then incubated in complete fresh media. After 24h CM were collected and assayed for osteoprotegerin (OPG) (R&D Systems, Minneapolis, MN), interleukin-6 (IL-6) (R&D Systems), and RANKL (PeproTech, Cranbury, NJ). Cell layers were lysed in 0.05% Triton X-100 and assayed for total DNA content (Promega QuantiFluor* dsDNA, VWR, Radnor, PA). Protein levels were normalized to total DNA content.

### Effect of estrogen on cancer cell proliferation and apoptosis

#### DNA synthesis (cell number; proliferation)

MDA-MB-231 cells were plated on 96–well plates and cultured until 80% confluence and then serum starved in media lacking FBS for 48 h. The cells were then treated with vehicle, 10^-8^ M, or 10^-7^ M E_2_ for 9 min and CM were replaced with fresh media for 20 h. After incubation, cells were pulsed with 10 μL/well of 1:1000 diluted 5-ethynyl-2′-deoxyuridine (EdU) and incubated at 37°C for another 4 h. Finally, cells were harvested and assessed for DNA synthesis as an indicator of proliferation according to the manufacturer’s protocol (Thermo Fisher Scientific).

#### Total p53 content

MDA-MB-231 cells were plated on 24–well plates and cultured to confluence, at which point the cells were treated with vehicle, 10^-8^ M, or 10^-7^ M E_2_ for 9 min and then incubated in fresh complete media. After 24 h, cell monolayers were lysed in 500 μL 1 mM EDTA, 0.05% TritonX-100. Total p53 content was measured in the cell layer lysate by ELISA according to the manufacturer’s protocol (R&D Systems) and normalized to total protein content (Pierce 660 nM BCA Protein Assay, Thermo Fisher Scientific).

#### Terminal Deoxynucleotidyl Transferase dUTP Nick End Labeling (TUNEL)

MDA-MB-231 cells were cultured in 96–well plates, treated with vehicle, 10^-8^ M, or 10^-7^ M E_2_ for 9 min, and then incubated in fresh complete media. After 24 h, cells were harvested and processed for TUNEL staining according to the manufacturer’s protocol (Trevigen, TiterTAC in situ microplate TUNEL assay).

### PLC and PKC signaling

Previous studies have shown that 17β-estradiol can regulate metastasis and invasion through a rapid ERα36–dependent membrane–mediated pathway involving PLC and PKC.^10^^,^^16^^,^^19^^,^^23^^,^^25–27^ To investigate if these signaling pathways are involved in the effect of E_2_ on TNBC osteolysis, we evaluated the activity of both PLC and PKC after treatment with E_2_ and E_2_-BSA. MDA-MB-231 cells were cultured to confluence on six–well plates and treated with vehicle, 10^-8^ M, or 10^-7^ M E_2_-BSA for 9 min. Cells were harvested immediately in 300 μL PLC lysis buffer or PKC lysis buffer according to the manufacturer’s protocol and assayed using the Amplex Red Phospholipase C Assay Kit (Thermo Fisher Scientific) or PKC Activity Assay Kit (Abcam), respectively. Cell layer lysates were assayed for total protein content using the Pierce BCA reagent kit (Thermo Fisher Scientific). PLC and PKC activities were normalized to total protein content per minute.

### *Effect of estrogen and TAM on TNBC osteolysis* in vivo

TAM is a common drug used in ER+ breast cancer cells. It competitively binds to ERα66, blocking E_2_ binding to the receptor, thereby inhibiting the tumorigenic effect of estrogen. It is unclear if TAM can act via the splice variant receptor ERα36. To investigate this question, a total of 48 non–ovariectomized 6–week–old female Foxn1^nu^ mice (*n* = 12/group) were separated into four groups: no treatment, TAM, 17β-estradiol, or a TAM/17β-estradiol combination group. During a premenopausal woman’s menstrual cycle, circulating estrogen changes, thereby exposing the cancer cells to various levels of estradiol. In healthy patients, the cancer cells are already accustomed to the fluctuation in estrogen. This study aimed to mimic premenopausal patients who receive hormone therapy like TAM without consideration of their systemic estrogen concentration.

All groups received implantation of MDA-MB-231 cells as previously described.^33^ Osmotic pumps (Innovative Research of America) delivering a 60–day release of 17β-estradiol and a matrix placebo (0.72 mg E2/d) or a 60–day release of TAM with a matrix placebo (5 mg E2/d), or a combination of both 17β-estradiol and TAM (17.5 mg E2 + 5 mg TAM/d), were placed subcutaneously between the scapulae of each animal at the time of cell implantation. After 8 weeks, all mice were euthanized and their legs were harvested for μCT and histological analysis as described previously.^33^

### Statistical analysis

Statistical significance between treatment legs and control (contralateral) legs was calculated by Wilcoxon matched–pairs signed rank test with *P* values ≤.05 considered significant. Differences between groups were determined by one–way ANOVA with a Bonferroni posttest. Quantitative analyses of in vitro studies are presented as means ± standard deviations. GraphPad was used to perform the Grubb’s outlier test to exclude outliers. Statistically significant differences between groups were determined by one–way ANOVA with a Bonferroni posttest (unless specified otherwise) with *P* values ≤ .05 considered significant.

## Results

### ER characterization

**Figure 1:**
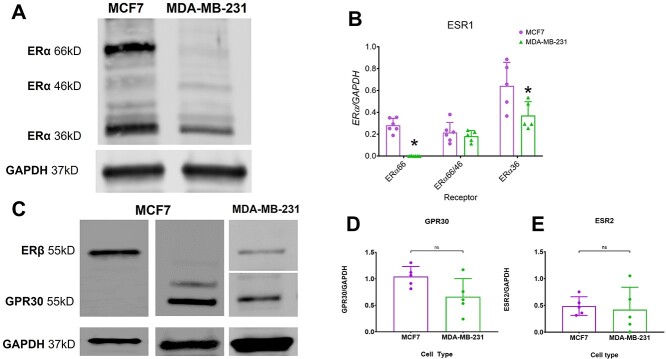
The expression and protein levels of ERs of MCF7 and MDA-MB-231. Baseline protein levels of ERα66, ERα46, and ERα36 in MDA-MB-231 cells assessed by western blot using GAPDH as the loading control (A). RNA expression levels of ERα66, ERα66/46, and ERα36 were measured by qPCR (B). Primers for ERα66 amplified exon 1 of the ESR1 gene, whereas the ERα66/46 and ERα36 primers amplified exons 7/8 and 9, respectively. Gene expression was normalized to GAPDH expression. Western blot analysis of ERβ and G–protein–coupled receptor 30 (GPR30) relative to GAPDH (C). Gene expression analysis of ESR2 (ERβ) and GPR30 (D). Data are presented as the mean ± standard deviation of *n* = 6 per cell type. Western blot image is a representative image of *n* = 3 per cell type. Groups labeled with and asterisk are statistically different compared with MCF7 receptor expression with *P* values ≤ .05 considered significant by one–way ANOVA with a Bonferroni posttest or a Student’s *t*-test. Data shown are representative of two independent studies.

Western blot analysis showed that ERα66, ERα46, and ERα36 were present in MCF7 cells, whereas only ERα46 and ERα36 were present in MDA-MB-231 cells (Figure 1A, Supplementary Figure S4). Gene expression confirmed these results. A primer designed to target both ERα66 and ERα46 indicated that both MCF7 and MDA-MB-231 cells positively expressed a combination of these receptors as well as ERα36; however, primers targeting ERα66 alone confirmed expression in MCF7 cells and a lack of expression in MDA-MB-231 cells (Figure 1B). Both MCF7 and MDA-MB-231 cells also possess ERβ and GPR30, which was confirmed by western blot and mRNA expression (Figure 1C-E, Supplementary Figures S5 and S6).

### Effect of estrogen in the ovariectomized osteolytic mouse model

**Figure 2:**
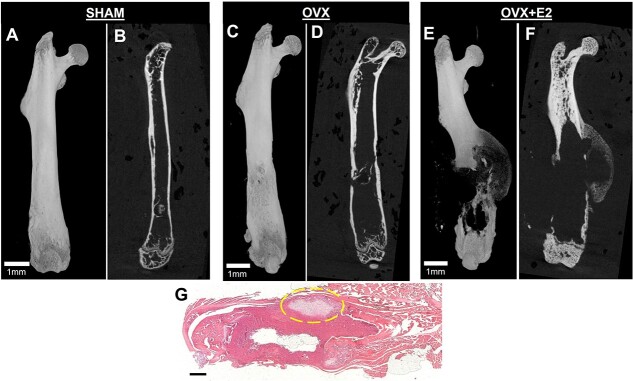
The effects of estrogen in ovariectomized mice. Representative 3D microCT (A) and 3D microCT (B) images of femurs from sham ovariectomized mice (*n* = 7) implanted with MDA-MB-231 cells into the bone marrow cavity. Representative 3D microCT (C) and 2D microCT (D) images of femurs from ovariectomized mice (*n* = 8) implanted with MDA-MB-231 cells. Representative 3D microCT (E) and 2D (F) images of femurs from ovariectomized mice exposed to estradiol treatment (*n* = 8). Representative hematoxylin and eosin–stained image of a femur from an ovariectomized mouse. Circle indicates osteolytic tumor resorbing cortical bone (G). Scale bar indicates 1 mm.

Eight weeks after implantation of cancer cells into ovariectomized mice, osteolytic tumors were evaluated by microCT and histology. Representative 2D and 3D microCT images showed that osteolytic tumors were not observed in the majority of sham and OVX mice but were present in the majority of estrogen treated OVX mice, which was confirmed histologically (Figure 2). The 2D microCT image was used to examine osteolysis by bisecting the bone. The 3D reconstruction illustrates the extent of extracortical tumor growth and resulting pathologic fracture if any. The imaging showed intact bone structure in the distal end of the femur in the sham and ovariectomized groups, respectively (Figure 2A-D). Ovariectomized mice treated with E_2_ had larger tumors that resorbed the bone as shown by the representative 2D and 3D images (Figure 2E and F). Histology images showed invasion of the bone by the tumor (Figure 2G).

**Figure 3:**
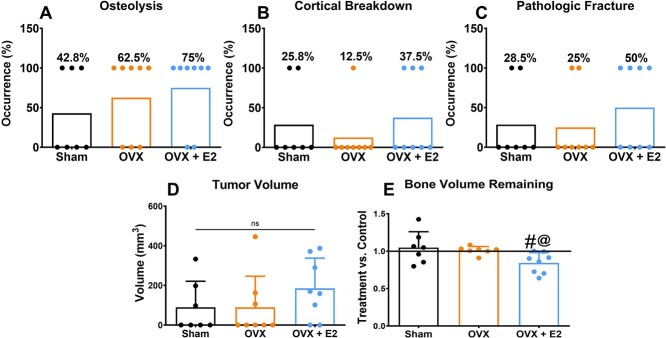
MicroCT analysis in ovariectomized and sham mice. Percent occurrence of osteolysis (A), breakdown of cortical bone (B), and pathological fracture (C) observed in sham (*n* = 7), OVX (*n* = 8), and OVX+ E_2_ (*n* = 8). Tumor volume comparing sham, ovariectomized, and ovariectomized mice exposed to estradiol (D). Groups not sharing a letter are statistically significant by one–way ANOVA with *P* values ≤ .05 considered significant. Bone volume remaining of treatment leg versus contralateral/control leg (E). Groups labeled with “#” are statistically significant compared with their respective control leg by Wilcoxon paired *t*-test with *P* values ≤ .05 considered significant. Groups labeled with an “@” are statistically significant by one–way ANOVA with a Bonferroni posttest and *P* values ≤ .05 considered significant.

The osteolytic lesions were observed in three out of a total of seven mice (42.8%) in the sham group, and increased to five mice out of eight (62.5%) in the ovariectomized group and six out of eight mice (75%) in the E_2_–treated ovariectomized group (Figure 3A). Breakdown of the cortical bone was detected in 25.8% of the mice with sham femurs, whereas only 12.5% of the mice in ovariectomized group exhibited cortical bone lesions. In contrast, 37.5% of the ovariectomized mice treated with E_2_ experienced cortical bone breakdown (Figure 3B). Pathological fractured femurs were observed in almost twice the number of the mice that were ovariectomized and treated with E_2_ compared with those in the sham and ovariectomized only groups (Figure 3C). There was no difference in the tumor volume across all groups (Figure 3D). Compared with their respective contralateral legs, the group subjected to estradiol had significantly less bone volume remaining at harvest. There was no significant difference between the bone volume remaining in either the sham or the ovariectomized femurs compared with their respective contralateral legs. However, when compared with the tumor cell bearing femurs in the sham group, the estradiol–treated ovariectomized group femurs had significantly less bone volume when comparing the difference in bone volume remaining to each group’s respective contralateral leg (Figure 3E).

### Effect of estrogen on breast cancer cell and osteoclast cross-talk

**Figure 4:**
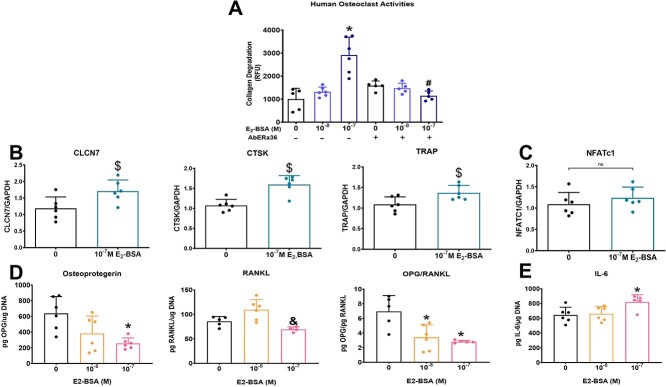
Estrogen increased cancer cell–induced osteoclast activation. Measurement of osteoclast activation assessed as collagen degradation by human osteoclast precursor cells after treatment with ERα36 antibody and E_2_ conjugated to BSA. Groups labeled with an asterisk are statistically significant compared with no treatment control at *P* < .05 by one–way ANOVA, whereas groups labeled with “#” are statistically significant compared with their respective E_2_-BSA treatment (A). RAW264.7 cell mRNA expression of CLCN7, CTSK, and TRAP (B) and NFATc1 (C) after treatment with MDA-MB-231 cell CM for 48 h. Groups labeled with “$” are statistically significant compared with control with *P* values ≤ .05 considered significant by Student’s *t*-test. Protein levels of OPG, RANKL, OPG/RANKL ratio (D), and IL-6 (E) secreted by MDA-MB-231 cells after E_2_-BSA. Groups labeled with an asterisk are statistically significant with *P* values ≤ .05 considered significant compared with control by one–way ANOVA with a Bonferroni posttest. Groups labeled with a “&” are statistically significant with *P* values ≤ .05 considered significant compared with the 10^-8^ M group by one–way ANOVA with a Bonferroni posttest data shown are representative of two independent studies.

To assess the effect of membrane impermeable E_2_-BSA on cancer cells’ ability to induce osteoclast activation and differentiation, MDA-MB-231 cells were treated with E_2_-BSA, and the CM were used to treat osteoclast precursor cells (Figure 4). CM from MDA-MB-231 cells after treatment with only the highest concentration of E_2_-BSA (10^-7^ M) enhanced the degradation of collagen thereby increasing the activation of the osteoclast precursor cells. The lower concentrations of E_2_-BSA (10^-8^ M) had no effect on collagen degradation compared with the control. However, CM from MDA-MB-231 cells treated with an antibody against ERα36 concurrently with treatment with 10^-7^ M E_2_-BSA, significantly reduced the level of collagen degradation by the human OCPs compared with treatment with 10^-7^ M E_2_-BSA (Figure 4A).

RAW264.7 murine macrophage cells were used in a separate experiment to investigate the effect of E_2_-BSA on the interaction of MDA-MB-231 cells and osteoclasts. The cells were first treated with 100 ng/mL RANKL to induce osteoclast differentiation. These cells were then treated with CM from MDA-MB-231 cells treated with or without E_2_-BSA. After 48 h, in response to CM from 10^-7^ M E_2_-BSA–treated MDA-MB-231 cells, RAW264.7 cells exhibited an increase in mRNA for the chloride voltage–gated channel-7 (CLCN7), which is the pH chloride channel use by osteoclasts to create an acidic environment to resorb bone (Figure 4B). mRNAs for cysteine proteinase cathepsin k (CTSK) (Figure 4B) and tartrate–resistant acid phosphatase (TRAP), the bone resorption marker (Figure 4B), were also increased. There was no effect on the master transcription factor nuclear factor of activated T cells 1 (NFATC1) after 48 h of treatment (Figure 4C). However, CM treatment for 24 h increased RAW264.7 NFATc1 expression but had no effect on CLCN7, TRAP, or CTSK mRNA (Supplementary Figure S7).

OPG is a soluble RANKL decoy receptor secreted by osteoblasts to prevent osteoclast formation and resorption. Treatment with the highest concentration of E_2_-BSA reduced MDA-MB-231 cell production of OPG (Figure 4D). The literature has shown that RANKL regulates osteoclast formation and activation. E_2_-BSA increased the production of RANKL in a biphasic manner with the maximum production at the 10^-8^ M concentration and was reduced at the 10^-7^ M (Figure 4D). The ratio of OPG to RANKL production was reduced after treatment with E_2_-BSA (Figure 4D). The 10^-7^ M E_2_-BSA treatment increased the production of IL-6 by MDA-MB-231 cells compared with vehicle (Figure 4E).

### Effect of estrogen on cancer cell proliferation and apoptosis

**Figure 5:**
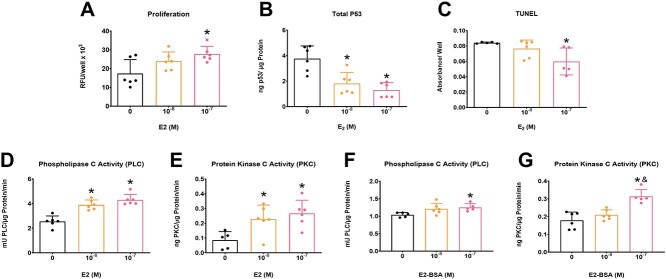
Estrogen regulates cancer cell proliferation and apoptosis involving PLC and PKC signaling pathways. MDA-MB-231 cells treated with vehicle, 10^-8^ M E_2_, or 10^-7^ M E_2_ and evaluated for proliferation via EdU assay (A), and apoptosis via p53 (B) or TUNEL (C). PLC activity (D) and PKC activity (E) after treatment with E_2_. PLC activity (F) and PKC activity (G) after treatment with E_2_-BSA. Groups labeled with an asterisk are statistically significant compared with vehicle with *P* values ≤ .05 considered significant by one–way ANOVA with a Bonferroni posttest. Groups labeled with a “&” are statistically significant compared with the 10^-8^ M group with *P* values ≤ .05 considered significant by one–way ANOVA with a Bonferroni posttest. Data shown are representative of two independent studies.

Estrogen increased MDA-MB-231 proliferation at the highest concentration (Figure 5A). All treatments with estrogen reduced total p53 content (Figure 5B) but only the highest concentration of estrogen reduced TUNEL (Figure 5C).

### Effect of estrogen on PLC and PKC activity

E_2_ treatment (10^-8^ M and 10^-7^ M) increased PLC– and PKC–specific activity in MDA-MB-231 cells (Figure 5D and E). Membrane impermeable E_2_-BSA increased PLC activity only at the higher concentration of E_2_-BSA compared with vehicle (Figure 5F), whereas the highest concentration increased PKC activity compared with both vehicle and the 10^-8^ M group (Figure 5G).

### Effect of estrogen and TAM on osteolysis in vivo

**Figure 6:**
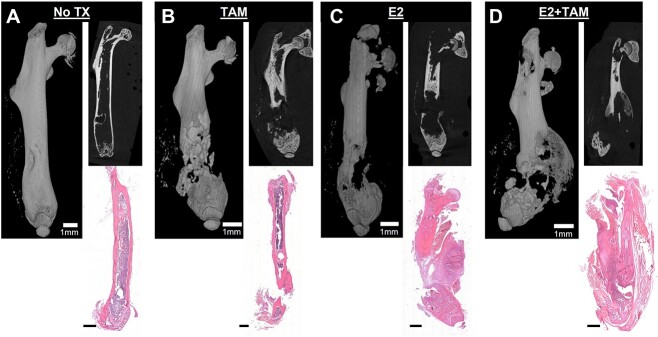
The effect of TAM and estrogen on osteolysis. Representative 3D (left) microCT images, 2D (right) microCT images, and hematoxylin and eosin stain image of femurs from non–treated mice (*n* = 11) (A). Representative 3D (left) microCT images, 2D (right) microCT images, and hematoxylin and eosin stain image of femurs from TAM treated mice (*n* = 12) (B). Representative 3D (left) microCT images, 2D (right) microCT images, and hematoxylin and eosin stain image of femurs from estrogen treated mice (*n* = 12) (C). Representative 3D (left) microCT images, 2D (right) microCT images, and hematoxylin and eosin stain image of femurs from the combine estrogen and TAM treated mice (*n* = 12) (D). Scale bar indicates 1 mm.

**Figure 7:**
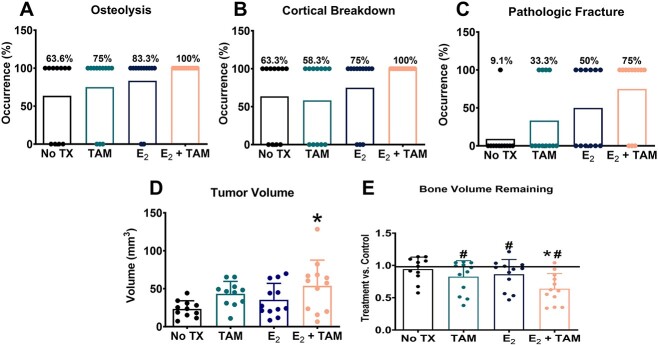
TAM combined with estrogen increase osteolysis. MicroCT analysis. Percent occurrence of osteolysis (A), breakdown of cortical bone (B), and pathological fracture (C) observed in no treatment (*n* = 11), TAM (*n* = 12), estradiol (E_2_) (*n* = 12), and the combination of estradiol and TAM (E_2_ + TAM). Tumor volume comparing no treatment, TAM, estradiol, and the combination of estradiol and TAM (D). Groups not sharing a letter are statistically significant by one–way ANOVA with *P* values ≤ .05 considered significant. Bone volume remaining of treatment leg versus contralateral/control leg (E). Groups labeled with “#” are statistically significant compared with their respective control leg by Wilcoxon paired *t*-test with *P* values ≤ .05 considered significant. Groups labeled with an asterisk are statistically significant compared with No treatment by one–way ANOVA with *P* values ≤ .05 considered significant.

MicroCT imaging showed prominent tumor development in the TAM, E_2_, and combination E_2_/TAM groups. The group that did not receive treatment experienced the least tumor burden with little to no tumor development as shown by the representative 3D and 2D microCT images as well as the histological image (Figure 6A). One mouse was excluded from the no treatment group because of death in the 1st week of the study. The TAM group developed tumors resulting in fractured femurs shown by representative microCT and histology images (Figure 6B). The estrogen–treated groups developed tumors similar to those of the TAM–treated groups. MicroCT and histological imaging showed tumors with highly visible fractures (Figure 6C). Large tumors were observed in the TAM/estrogen combination group. The representative microCT and histological images show tumors that have caused large scale fracturing (Figure 6D). About 63.6% of mice from the no treatment group developed osteolytic tumors, whereas tumors in the TAM and E_2_–treated groups developed in 75% or 83.3% of the mice, respectively. All femurs of the E_2_ and TAM combination treated mice developed tumors (Figure 7A). Breakdown of the cortical bone was detected in 63.3% of the no treatment femurs, whereas only 58.3% in TAM–treated mice. The breakdown of the cortical bone was observed in 75% of the E_2_–treated mice. Treatment with a combination of E_2_ and TAM subjected all femurs to cortical bone breakdown (Figure 7B). Pathological fractures were observed in only 9.1% of non–treated mice, whereas only one-third of the TAM–treated mice had femurs with a pathological fracture. Half of the group exposed to estradiol without TAM experienced fractures. In all, 75% of the mice receiving the combination of TAM and E_2_ had femurs that developed fractures (Figure 7C). There was no difference in the tumor volume of the TAM or the E_2_ groups compared with the no treatment control mice. However, the volume of the tumors measured in mice receiving the combination of E_2_ and TAM was significantly larger than the control (Figure 7D). The bone volume remaining in the leg receiving tumor cells was compared with the contralateral control leg. The TAM, E_2_, and combination of E_2_ and TAM groups all had reduced bone volume remaining compared with their respective contralateral legs. Only the combination treatment of E_2_ and TAM had significantly lower bone volume compared with the no treatment control (Figure 7E), when comparing the difference in bone volume remaining to each group’s respective contralateral leg.

## Discussion

Estrogen’s critical role in regulating tumorigenesis has made the canonical ER, ERα66, a crucial marker in diagnosis, prognosis, and therapeutics.^6–9^ These studies predate the identification of ER splice variants, raising the question of their role in mediating breast cancer tumorigenesis in TNBC. To address this, we took advantage of a bone interface model in mice using ERα66 negative MDA-MB-231 cells. We first confirmed that the cells express ERα36, ERβ, and GPR30. When we used a primer targeting ERα66 and ERα46, we observed amplification similar to that of MCF7 cells. However, using a primer targeting only ERα66, no expression of the larger form was observed compared with MCF7 cells. This indicates that MDA-MB-231 cells do not express ERα66 but do express ERα46, which was confirmed by western blot analysis. ERα36 was measured in both cell types, but levels of this ER isoform were higher in MCF7 cells. ERα46 is similar to ERα66 in its subcellular localization but does not contain the transactivation domain AF-1. Some studies suggest that ERα46 acts as a competitive inhibitor of ERα66; however, additional studies are necessary to investigate the role of ERα46 in mediating transcriptional effects of estrogen in disease progression.^46^^,^^47^ Estrogen mediates its genomic and non–genomic effects through the classical nuclear receptor, ERα66, and the membrane associated receptor, ERα36, respectively. The present study focused on the role that ERα36 plays in tumor induced osteolysis, but the role of ERα46 cannot be ruled out.

Athymic homozygous Foxn1^nu^ mice are a commonly used murine strain with a deletion of the Foxn1 gene, causing a deterioration of the thymus. This results in a diminished immune system based on a reduced of T cells, making these mice a desirable tool in cancer research. Ultimately, no animal model is without its limitations. Our previous work^33^ establishing this bone–tumor interface model using the aggressive, triple–negative MDA-MB-231 breast cancer cells produced consistent osteolysis, tumor formation, and pathologic fracture. We observed successful engraftment with no evidence of xenorejection, and therefore chose to use this model for the present studies. The aim of using this model was to evaluate the effects on bone osteolysis, a terminal stage of the bone metastatic process; therefore, we did not examine the effects of epithelial to mesenchymal transition nor the migration of the cells to the bone.

Because of the limitation of this model not accurately mimicking the environment of immunocompetent aging women or the process of bone metastasis in its entirety, further studies must be performed to validate these results in a clinical setting before making any major clinical decisions. The late endpoint at 8 weeks postimplantation also limited the ability to measure various cellular parameters because of the level of bone destruction at harvest. While an earlier time point may have allowed measurements of TRAP staining and other cellular parameters, the effects on tumor growth would not have been seen. The observance of osteolysis within the bone being a priority, the later time point was chosen.

Ovariectomized mice were used to determine if the formation of MDA-MB-231 tumors in bone was sensitive to E_2_. The ovariectomized mice showed no difference in tumor volume or bone volume remaining compared with the sham ovariectomy group or their respective contralateral legs, indicating that the loss of estrogen did not affect tumor burden. However, when treated with E_2_, the tumor further eroded the bone and increased the appearance of osteolytic lesions. This was shown by a decrease in the total bone volume remaining after 8 weeks of growth. However, the effect seen with E_2_ treatment was a sum of the effect of ovariectomy and cancer cell growth, which may explain the lack of changes to the bone volume remaining.

Taken together, these data suggest that estrogen increased TNBC–induced osteolysis and tumor burden. These results indicate that although the tumor did not possess canonical ERα66, it responded to estrogen to regulate bone osteolysis. Based on this observation, in addition to the aim of the study involving osteolysis and not metastasis, other organs were not assessed at the time of harvest. These findings not only demonstrate a change in the behavior of the tumor but suggest a combination of changes to both the tumor and the host tissue.

While this model mimics osteolytic breakdown of the bone because of tumor burden, it is not an exact indication of bone metastasis. In addition, using microCT to quantify extracortical tumor volume is not a perfect method. The damage to the bone makes it difficult to accurately distinguish the margins of the tumor or any further parameters, including TRAP staining. Thus, the standard deviations within each group are quite large, and statistically, no differences were observed. However, the main focus of our study was not to evaluate the change in tumor volume after treatment but to examine the effects on osteolysis; therefore, no other modalities of tumor volume measurements were performed. In order to further examine the effects of E_2_ on bone metastasis, a different model must be used.

E_2_ regulates MDA-MB-231–induced osteoclastogenesis via paracrine regulation of osteoclast formation and activity in vitro. Moreover, this effect observed is mediated through ERα36–dependent signaling pathways. We used E_2_ conjugated to BSA and a short exposure time (9 min) to observe the rapid membrane associated effects and limit E_2_ transport into the cells.^16^^,^^44^ In addition, cultures were co-treated with anti–ERα36 antibodies to block activation via that receptor. Finally, pre–osteoclasts and RAW264.7 cells were treated with CM generated by the ER- tumor cells. The results of the in vitro studies support the hypothesis that MDA-MB-231 cells exert their effects by producing factors that stimulate inflammation and osteoclast formation and are exacerbated by E_2_ via ERα36. Human OCPs treated with CM from MDA-MB-231 cells treated with E_2_-BSA increased collagen degradation, suggesting that the E_2_-BSA increased the production of factors that activated osteoclasts to resorb the bone. While it is possible that the residual unconjugated E_2_ present in the CM could have caused this effect on osteoclasts, treatment with E_2_-BSA in conjunction with an antibody to block ERα36 also blocked this effect. Inclusion of anti–ERα36 antibody restored collagen degradation to control levels, supporting the conclusion that ERα36 was responsible for the cancer cell–induced osteoclast activation.

These data confirm previous experiments in the literature evaluating osteoclast activity in postmenopausal women with normal versus significantly lower estrogen levels.^30^ ERα36 was highly expressed in osteoclasts of primary bone from normal estrogen postmenopausal women compared with osteoclasts found in the primary bone of postmenopausal women categorized with weak estrogen levels.^30^ Together, these results suggest that ERα36 is a critical player in the activation of osteoclasts with exposure to higher estrogen levels. These findings indicate that estrogen induces MDA-MB-231 cells to secrete factors that increase osteoclast activation and thereby increase collagen degradation. However, this experiment did not demonstrate which factors are being produced by the osteoclasts to increase matrix degradation.

These observations raised the question of what aspect of osteoclastogenesis was driven by E_2_ in TNBC cells. At 24 h, the master transcription gene NFATc1 was upregulated, but the other genes were unaffected. After 48 h of treatment with CM, CLCN7, which encodes CLCN7, and CTSK, which encodes CTSK, increased, indicating that these genes were regulated via ERα36 signaling through NFATc1. These data, along with previous data that demonstrate NFATc1 expression precedes RANKL–mediated osteoclastogenesis,^48–50^ suggest that E_2_-BSA treatment increased chloride channel secretion and matrix degradation proteinases, increasing the ability of osteoclasts to break down and resorb the bone.^47^ TRAP, a common bone resorption marker involved in osteoclast activity through the fusion of mononucleated pre-osteoclasts to multinucleated osteoclasts, was also increased.^51^ In all, these data suggest that membrane bound E_2_ plays a role in the activation of osteoclasts and the breakdown of the extracellular matrix of bone.

The increase in osteoclast activation markers after treatment with CM supports the role of MDA-MB-231–secreted proteins in driving bone resorption. Osteoblasts regulate osteoclast formation through the coordinated release of OPG and RANKL.^52^^,^^53^ OPG protects bone from excessive resorption by binding to RANKL and preventing it from binding to its receptor RANK on OCPs.^52–54^ E_2_-BSA treatment caused a reduction in OPG and upregulated RANKL, thereby reducing the OPG/RANKL ratio. The shift in RANKL expression may have been secondary to the upregulation of IL-6, which is known to increase RANKL expression.^52^^,^^54^ This suggests that E_2_-BSA induced the secretion of proteins by TNBC cells, which increased osteoclast differentiation and activity.

Our data indicated that cancer cells secrete proteins that increase the activation of osteoclasts to then break down bone extracellular matrix. This supports the hypothesis that it is the combined responses of cancer cells and host cells that increase osteolysis. To understand the role of cancer cells in the increase of osteolysis observed in vivo, we examined the mechanisms by which E_2_ modulates cancer cell proliferation and apoptosis in vitro. Treatment of the MDA-MB-231 cells with E_2_ increased DNA synthesis and reduced p53 and TUNEL, indicating that E_2_ increased cell growth and reduced cell apoptosis.

Previously, we showed that E_2_ can regulate metastasis through a membrane–associated signaling pathway mediated by PLC and PKC.^19^^,^^21^^,^^23^^,^^26^^,^^27^ This signaling pathway, which is conserved in other cells,^20^^,^^26–29^ involves E_2_–dependent activation of PKC via G–protein–coupled PLC that is regulated by a membrane–mediated mechanism independent of the classical ERα66 receptor.^16^^,^^28^^,^^55^ Inhibition of PLC and PKC reduced the effect of E_2_ on MDA-MB-231 cell metastasis, and the activity of both enzymes was activity by E_2_, suggesting that the mechanisms by which PLC and PKC are activated may also involve membrane–independent pathways.^21^^,^^23^^,^^25^^,^^55^^,^^56^ Previous studies from our lab demonstrated that E_2_ and E_2_-BSA activates PKCα and the translocation of PKCε across different cell types and species.^21^^,^^57^ While the experiments performed in the present study did not examine which PKC isoform(s) was activated by E_2_, similar mechanisms are likely involved.

As previous studies have shown, ERα36 overexpression results in a poorer prognosis and a more TAM–resistant tumor.^9^^,^^22^^,^^31^^,^^35^^,^^36^ The membrane–mediated pathway involving ERα36 may be the mechanism involved in acquiring TAM resistance.^16^ Our results showed that TAM could not block the reduction of bone volume in vivo. However, compared with the no treatment control, the TAM and estradiol groups were not significantly different. When TAM and estradiol were combined, the occurrence of osteolysis, breakdown of cortical bone, and pathological fractures were observed in every mouse. The volume of the tumor was significantly increased, and the remaining bone volume was also decreased. This was shown quantitatively through microCT and qualitatively through histological imaging. These data suggest that while MDA-MB-231 cells do not express the classical ERα66 receptor, the ERα36 receptor is still active and is unaffected by TAM treatment. TAM could not block the signaling pathway involving ERα36; the results indicate that the combination of estradiol and TAM increased bone lysis. This study aimed to mimic the effects of TAM treatment in premenopausal patients experiencing influxes in estradiol because of menstruation. These data suggest that TAM treatment may increase the risk of bone lysis during periods of increased estradiol levels.

Although our results confirmed previous studies showing that MDA-MB-231 cells do not express ERα66 but still express ERα36, our data also showed that these cells express ERα46, ERβ, and GPR30, which may be involved in modulating osteolysis.^58^ The roles of these receptors in E_2_–induced osteolysis were not ruled out, although it is unlikely that they contribute mechanistically to the ERα36–dependent effects based on our results using the anti–ERα36 antibody to block signaling by this receptor specifically.

In summary, the results of the present study demonstrated that estradiol enhanced TNBC (ERα66-, ERα36+), induced osteolytic lesions, and reduced the volume of bone remaining after resorption. In vitro results suggest that this response occurs via a rapid membrane–mediated signaling pathway that increases tumor growth and activates PLC and PKC to increase the production of osteoclast activating proteins RANKL and IL-6 to inhibit the production of OPG. Activated osteoclasts, in turn, increase the expression of matrix degradation proteases to resorb the bone. Our data suggest that without ERα66, E_2_ mediates the cancer cell–induced matrix degradation through ERα36. TAM enhanced the effect of estradiol on increasing osteolysis and tumor volume, indicating that it was involved with increased bone destruction and invasion. The lack of ERα66 in this cell model, and our in vitro results suggest that the mechanism of enhanced osteolysis involved a rapid membrane–associated pathway mediated by ERα36 expressed in the tumor. Future studies will seek to further elucidate these pathways.

## Supplementary Material

Supplementary_ziae041

## Data Availability

The data generated in this study are available upon reasonable request from the corresponding author.

## References

[1] Siegel RL , Miller KD, Fuchs HE, Jemal A. Cancer statistics, 2022. CA Cancer J Clin. 2022;72(1):7–33. 10.3322/caac.21708 35020204

[2] Howard FM , Olopade OI. Epidemiology of triple-negative breast cancer: a review. Cancer J. 2021;27(1):8–16. 10.1097/PPO.0000000000000500 33475288 PMC12050094

[3] Pulido C , Vendrell I, Ferreira AR, et al. Bone metastasis risk factors in breast cancer. Ecancermedicalscience. 2017;11:715. 10.3332/ecancer.2017.715 28194227 PMC5295847

[4] Coleman RE , Smith P, Rubens RD. Clinical course and prognostic factors following bone recurrence from breast cancer. Br J Cancer. 1998;77(2):336–340. 10.1038/bjc.1998.52 9461007 PMC2151240

[5] Cetin K , Christiansen CF, Sværke C, Jacobsen JB, Sørensen HT. Survival in patients with breast cancer with bone metastasis: a Danish population-based cohort study on the prognostic impact of initial stage of disease at breast cancer diagnosis and length of the bone metastasis-free interval. BMJ Open. 2015;5(4):e007702. 10.1136/bmjopen-2015-007702 PMC4420974 25926150

[6] Luo A , Wu F, Han R, et al. Clinicopathological features and prognostic evaluation of bone metastasis in triple-negative breast cancer. J Cancer Res Ther. 2017;13(5):778–784. 10.4103/jcrt.JCRT_543_17 29237903

[7] Jung EJ , Kim JY, Kim JM, et al. Positive estrogen receptor status is a poor prognostic factor in node-negative breast cancer: an observational study in Asian patients. Medicine (Baltimore). 2021;100(11):e25000. 10.1097/MD.0000000000025000 33725973 PMC7982180

[8] Howlader N , Altekruse SF, Li CI, et al. US incidence of breast cancer subtypes defined by joint hormone receptor and HER2 status. J Natl Cancer Inst. 2014;106(5):dju055. 10.1093/jnci/dju055 PMC4580552 24777111

[9] Sharma D , Saxena NK, Davidson NE, Vertino PM. Restoration of tamoxifen sensitivity in estrogen receptor-negative breast cancer cells: tamoxifen-bound reactivated ER recruits distinctive corepressor complexes. Cancer Res. 2006;66(12):6370–6378. 10.1158/0008-5472.CAN-06-0402 16778215 PMC2925469

[10] Chaudhri RA , Hadadi A, Lobachev KS, Schwartz Z, Boyan BD. Estrogen receptor-alpha 36 mediates the anti-apoptotic effect of estradiol in triple negative breast cancer cells via a membrane-associated mechanism. Biochim Biophys Acta. 2014;1843(11):2796–2806. 10.1016/j.bbamcr.2014.07.019 25108195 PMC4296672

[11] Brook N , Gill J, Chih H, et al. Pigment epithelium-derived factor downregulation in oestrogen receptor positive breast cancer bone metastases is associated with menopause. Mol Cell Endocrinol. 2023;559:111792. 10.1016/j.mce.2022.111792 36309204

[12] Yip CH , Rhodes A. Estrogen and progesterone receptors in breast cancer. Future Oncol. 2014;10(14):2293–2301. 10.2217/fon.14.110 25471040

[13] Li CH , Karantza V, Aktan G, Lala M. Current treatment landscape for patients with locally recurrent inoperable or metastatic triple-negative breast cancer: a systematic literature review. Breast Cancer Res. 2019;21(1):143. 10.1186/s13058-019-1210-4 31842957 PMC6916124

[14] Wang ZY , Yin L. Estrogen receptor alpha-36 (ER-α36): a new player in human breast cancer. Mol Cell Endocrinol. 2015;418:193–206. 10.1016/j.mce.2015.04.017 25917453

[15] Park M , Lee SH, Bui QT, Kim YM, Kang KW. The essential role of YAP in ERα36-mediated proliferation and the epithelial-mesenchymal transition in MCF-7 breast cancer cells. Front Pharmacol. 2022;13:1057276. 10.3389/fphar.2022.1057276 36534032 PMC9755719

[16] Segars JH , Driggers PH. Estrogen action and cytoplasmic signaling cascades. Part I: membrane-associated signaling complexes. Trends Endocrinol Metab. 2002;13(8):349–354. 10.1016/S1043-2760(02)00633-1 12217492 PMC4137481

[17] Chaudhri RA , Schwartz N, Elbaradie K, Schwartz Z, Boyan BD. Role of ERα36 in membrane-associated signaling by estrogen. Steroids. 2014;81:74–80. 10.1016/j.steroids.2013.10.020 24252378

[18] Razandi M , Pedram A, Levin ER. Plasma membrane estrogen receptors signal to anti apoptosis in breast cancer. Mol Endocrinol. 2000;14(9):1434–1447. 10.1210/mend.14.9.0526 10976921

[19] Chaudhri RA , Olivares-Navarrete R, Cuenca N, Hadadi A, Boyan BD, Schwartz Z. Membrane estrogen signaling enhances tumorigenesis and metastatic of breast cancer cells via estrogen receptor-α36 (ERα36). J Biol Chem. 2012;287(10):7169–7181. 10.1074/jbc.M111.292946 22247547 PMC3293594

[20] Sylvia VL , Boyan BD, Dean DD, Schwartz Z. The membrane effects of 17beta-estradiol on chondrocyte phenotypic expression are mediated by activation of protein kinase C through phospholipase C and G-proteins. J Steroid Biochem Mol Biol. 2000;73(5):211–224. 10.1016/S0960-0760(00)00078-9 11070350

[21] Boyan BD , Sylvia VL, Frambach T, et al. Estrogen-dependent rapid activation of protein kinase C in estrogen receptor-positive MCF-7 breast cancer cells and estrogen receptor-negative HCC38 cells is membrane-mediated and inhibited by tamoxifen. Endocrinol. 2003;144(5):1812–1824. 10.1210/en.2002-221018 12697687

[22] Wang Q , Jiang J, Ying G, et al. Tamoxifen enhances stemness and promotes metastasis of ERα36+ breast cancer by upregulating ALDH1a1 in cancer cells. Cell Res. 2018;28(3):336–358. 10.1038/cr.2018.15 29393296 PMC5835774

[23] Schwartz N , Verma A, Bivens CB, Schwartz Z, Boyan BD. Rapid steroid hormone actions via membrane receptors. Biochim Biophys Acta. 2016;1863(9):2289–2298. 10.1016/j.bbamcr.2016.06.004 27288742

[24] Weitsman G , Lawler K, Kelleher MT, et al. Imaging tumour heterogeneity of the consequences of a PKCα-substrate interaction in breast cancer patients. Biochem Soc Trans. 2014;42(6):1498–1505. 10.1042/BST20140165 25399560 PMC4259014

[25] Lim PS , Sutton CR, Rao S. Protein kinase C in the immune system: from signalling to chromatin regulation. Immunology. 2015;146(4):508–522. 10.1111/imm.12510 26194700 PMC4693901

[26] Schwartz Z , Sylvia VL, Luna MH, et al. The effect of 24R,25-(OH)2D3 on protein kinase C activity in chondrocytes is mediated by phospholipase D whereas the effect of 1alpha,25-(OH)2D3 is mediated by phospholipase C. Steroids. 2001;66(9):683–694. 10.1016/S0039-128X(01)00100-3 11546556

[27] Boyan BD , Schwartz Z. Rapid vitamin D-dependent PKC signaling shares features with estrogen-dependent PKC signaling in cartilage and bone. Steroids. 2004;69(8-9):591–597. 10.1016/j.steroids.2004.05.008 15288775

[28] Sylvia VL , Walton J, Lopez D, Dean DD, Boyan BD, Schwartz Z. 17 beta-estradiol-BSA conjugates and 17 beta-estradiol regulate growth plate chondrocytes by common membrane associated mechanisms involving PKC dependent and independent signal transduction. J Cell Biochem. 2001;81(3):413–429. 10.1002/1097-4644(20010601)81:3<413::AID-JCB1055>3.0.CO;2-M 11255224

[29] Sylvia VL , Hughes T, Dean DD, Boyan BD, Schwartz Z. 17beta-estradiol regulation of protein kinase C activity in chondrocytes is sex-dependent and involves nongenomic mechanisms. J Cell Physiol. 1998;176(2):435–444. 10.1002/(SICI)1097-4652(199808)176:2<435::AID-JCP22>3.0.CO;2-0 9648931

[30] Xie H , Sun M, Liao XB, et al. Estrogen receptor α36 mediates a bone-sparing effect of 17β-estradiol in postmenopausal women. J Bone Miner Res. 2010;26(1):156–168. 10.1002/jbmr.169 PMC3179309 20578216

[31] Shi L , Dong B, Li Z, et al. Expression of ER-alpha36, a novel variant of estrogen receptor alpha, and resistance to tamoxifen treatment in breast cancer. J Clin Oncol. 2009;27(21):3423–3429. 10.1200/JCO.2008.17.2254 19487384 PMC2717750

[32] Finnegan RM , Elshazly AM, Patel NH, et al. The BET inhibitor/degrader ARV-825 prolongs the growth arrest response to fulvestrant + palbociclib and suppresses proliferative recovery in ER-positive breast cancer. Front Oncol. 2023;12:966441. 10.3389/fonc.2022.966441 36741704 PMC9890056

[33] Cohen DJ , Patel V, Verma A, Boyan BD, Schwartz Z. Effect of 17β-estradiol on estrogen receptor negative breast cancer cells in an osteolytic mouse model. Steroids. 2019;142:28–33. 10.1016/j.steroids.2017.10.010 29133279

[34] Zhao Y , Deng C, Lu W, et al. Let-7 microRNAs induce tamoxifen sensitivity by downregulation of estrogen receptor α signaling in breast cancer. Mol Med. 2011;17(11-12):1233–1241. 10.2119/molmed.2010.00225 21826373 PMC3321804

[35] Keene S , Azuelos C, Majumdar S. Sensitivity evaluation of two human breast cancer cell lines to tamoxifen through apoptosis induction. Open J Apoptosis. 2012;3(4):70–77. 10.4236/ojapo.2014.34008

[36] Razandi M , Pedram A, Jordan V, Fuqua S, Levin ER. Tamoxifen regulates cell fate through mitochondrial estrogen receptor beta in breast cancer. Oncogene. 2013;32(27):3274–3285. 10.1038/onc.2012.335 22907432 PMC3505272

[37] Ejaz I , Javed MA, Jan MS, et al. Rational design, synthesis, antiproliferative activity against MCF-7, MDA-MB-231 cells, estrogen receptors binding affinity, and computational study of indenopyrimidine-2, 5-dione analogs for the treatment of breast cancer. Bioorg Med Chem Lett. 2022;64:128668. 10.1016/j.bmcl.2022.128668 35276362

[38] Faul F , Erdfelder E, Lang AG, Buchner A. G*power 3: a flexible statistical power analysis program for the social, behavioral, and biomedical sciences. Behav Res Methods. 2007;39(2):175–191. 10.3758/BF03193146 17695343

[39] Wright LE , Ottewell PD, Rucci N, et al. Murine models of breast cancer bone metastasis. Bonekey Rep. 2016;5:804. 10.1038/bonekey.2016.31 27867497 PMC5108088

[40] Xu L , Wu Z, Zhou Z, Yang X, Xiao J. Intratibial injection of patient-derived tumor cells from giant cell tumor of bone elicits osteolytic reaction in nude mouse. Oncol Lett. 2018;16(4):4649–4655. 10.3892/ol.2018.9148 30214599 PMC6126149

[41] Cutrera J , Johnson B, Ellis L, Li S. Intraosseous inoculation of tumor cells into bone marrow promotes distant metastatic tumor development: a novel tool for mechanistic and therapeutic studies. Cancer Lett. 2013;329(1):68–73. 10.1016/j.canlet.2012.10.022 23111105 PMC3535523

[42] Park SH , Eber MR, Shiozawa Y. Models of prostate cancer bone metastasis. Methods Mol Biol. 2019;1914:295–308 30729472 10.1007/978-1-4939-8997-3_16 PMC6738334

[43] Willekens I , Buls N, Lahoutte T, et al. Evaluation of the radiation dose in micro-CT with optimization of the scan protocol. Contrast Media Mol Imaging. 2010;5(4):201–207. 10.1002/cmmi.394 20665903

[44] Zheng J , Ali A, Ramirez VD. Steroids conjugated to bovine serum albumin as tools to demonstrate specific steroid neuronal membrane binding sites. J Psychiatry Neurosci. 1996;21(3):187–197 8935331 PMC1188766

[45] Stevis PE , Deecher DC, Suhadolnik L, Mallis LM, Frail DE. Differential effects of estradiol and estradiol-BSA conjugates. Endocrinol. 1999;140(11):5455–5458. 10.1210/endo.140.11.7247 10537181

[46] Figtree GA , McDonald D, Watkins H, Channon KM. Truncated estrogen receptor alpha 46-kDa isoform in human endothelial cells: relationship to acute activation of nitric oxide synthase. Circulation. 2003;107(1):120–126. 10.1161/01.CIR.0000043805.11780.F5 12515753

[47] Flouriot G , Brand H, Denger S, et al. Identification of a new isoform of the human estrogen receptor-alpha (hER-alpha) that is encoded by distinct transcripts and that is able to repress hER-alpha activation function 1. EMBO J. 2000;19(17):4688–4700. 10.1093/emboj/19.17.4688 10970861 PMC302047

[48] Takayanagi H , Kim S, Koga T, et al. Induction and activation of the transcription factor NFATc1 (NFAT2) integrate RANKL signaling in terminal differentiation of osteoclasts. Dev Cell. 2002;3(6):889–901. 10.1016/S1534-5807(02)00369-6 12479813

[49] Matsumoto M , Kogawa M, Wada S, et al. Essential role of p38 mitogen-activated protein kinase in cathepsin K gene expression during osteoclastogenesis through association of NFATc1 and PU.1. J Biol Chem. 2004;279(44):45969–45979. 10.1074/jbc.M408795200 15304486

[50] Kim K , Kim JH, Lee J, et al. Nuclear factor of activated T cells c1 induces osteoclast-associated receptor gene expression during tumor necrosis factor-related activation-induced cytokine-mediated osteoclastogenesis. J Biol Chem. 2005;280(42):35209–35216. 10.1074/jbc.M505815200 16109714

[51] Xing L , Xiu Y, Boyce BF. Osteoclast fusion and regulation by RANKL-dependent and independent factors. World J Orthop. 2012;3(12):212–222. 10.5312/wjo.v3.i12.212 23362465 PMC3557323

[52] Boyce BF , Xing L. Functions of RANKL/RANK/OPG in bone modeling and remodeling. Arch Biochem Biophys. 2008;473(2):139–146. 10.1016/j.abb.2008.03.018 18395508 PMC2413418

[53] Marcadet L , Bouredji Z, Argaw A, Frenette J. The roles of RANK/RANKL/OPG in cardiac, skeletal, and smooth muscles in health and disease. Front Cell Dev Biol. 2022;10:903657. 10.3389/fcell.2022.903657 35693934 PMC9181319

[54] Chowdhury S , Schulz L, Palmisano B, et al. Muscle-derived interleukin 6 increases exercise capacity by signaling in osteoblasts. J Clin Invest. 2020;130(6):2888–2902. 10.1172/JCI133572 32078586 PMC7260002

[55] Igumenova TI . Dynamics and membrane interactions of protein kinase C. Biochemistry. 2015;54(32):4953–4968. 10.1021/acs.biochem.5b00565 26214365 PMC4979571

[56] Martelli AM , Evangelisti C, Nyakern M, Manzoli FA. Nuclear protein kinase C. Biochim Biophys Acta. 2006;1761(5-6):542–551. 10.1016/j.bbalip.2006.02.009 16574477

[57] Lavie Y , Zhang ZC, Cao HT, et al. Tamoxifen induces selective membrane association of protein kinase C epsilon in MCF-7 human breast cancer cells. Int J Cancer. 1998;77(6):928–932. 10.1002/(SICI)1097-0215(19980911)77:6<928::AID-IJC22<3.0.CO;2-W 9714066

[58] Hinsche O , Girgert R, Emons G, Gründker C. Estrogen receptor β selective agonists reduce invasiveness of triple-negative breast cancer cells. Int J Oncol. 2015;46(2):878–884. 10.3892/ijo.2014.2778 25420519

