# Supplementary material for: Estradiol induces bone osteolysis in triple–negative breast cancer via its membrane–associated receptor ERα36
Source: JBMR Plus. 2024 Mar 26;8(5):ziae041. doi: 10.1093/jbmrpl/ziae041 (PMC11032217; doi:10.1093/jbmrpl/ziae041)
Supplement: Supplementary_ziae041 [file supplementary_ziae041.docx]

**Supplementary Tables**

Supplemental Table 1: Gene specific sequences of quantitative PCR primers

| **Genes** | **Primer Sequences** | |
| --- | --- | --- |
| GAPDH | Forward | TGTTCCTACCCCCAATGTGT |
|  | Reverse | CTCAGATGCCTGCTTCACC |
| ERa66 | Forward | TGCCTGGAGTGATGTTTAAGC |
|  | Reverse | ACGGGAGCAAGTGCAGTC |
| ERa36 | Forward | TCCTCGTGTCTAAAGCCTCTG |
|  | Reverse | AAAATGTCCCCACGTCCACA |
| ERa66/46 | Forward | TGCGTCGCCTCTAACCTCG |
|  | Reverse | TCCCAGATGCTTTGGTGTGG |
| ESR2 | Forward | CCTCCTATGTAGACAGCCACCA |
|  | Reverse | TGGCGCAACGGTTCCCACTAA |
| GPR30 | Forward | TTCAGCAGTGCCGTGTAGA |
|  | Reverse | GTGTGCAGCTCCCGAGTC |
| NFATc1 | Forward | CCGTTGCTTCCAGAAAATAACA |
|  | Reverse | TGTGGGATGTGAACTCGGAA |
| CTSK | Forward | ATCTTGTGGACTGTGTGACTGAGAATTA |
|  | Reverse | GCCGTGGCGTTATACATACAACTT |
| TRAP | Forward | GGC TAC TTG CGG TTT CAC TA |
|  | Reverse | CTT GGG AGG CTG GTC TTA AA |
| CLCN7 | Forward | CTGTGATCGTGGCCTTCATA |
|  | Reverse | CTTGATCACCAGCGTCTTGA |


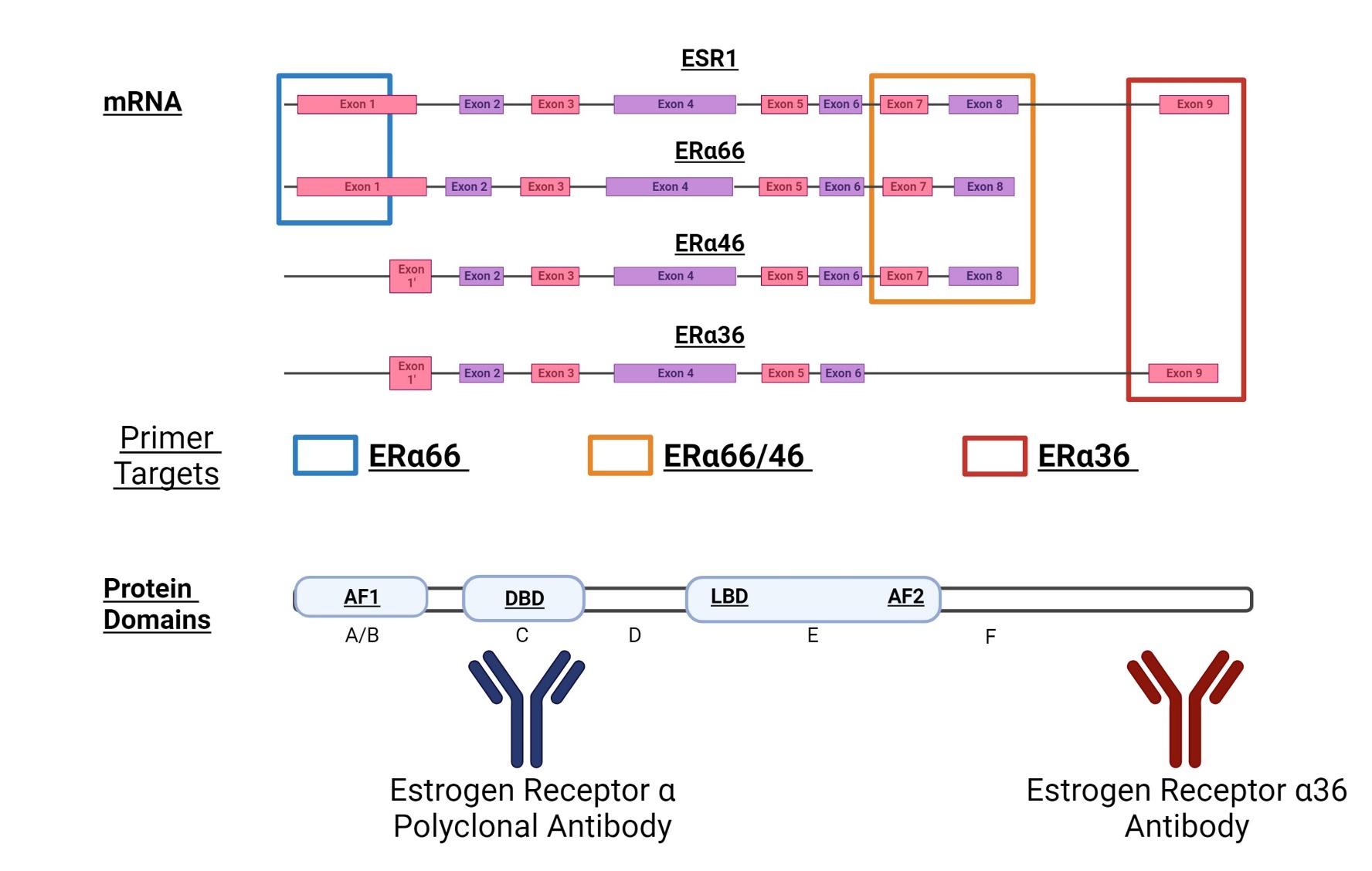
Supplemental Figure 1

**Supplemental Figure 1: Schematic of Estrogen Receptor alpha primer and antibody targets.**


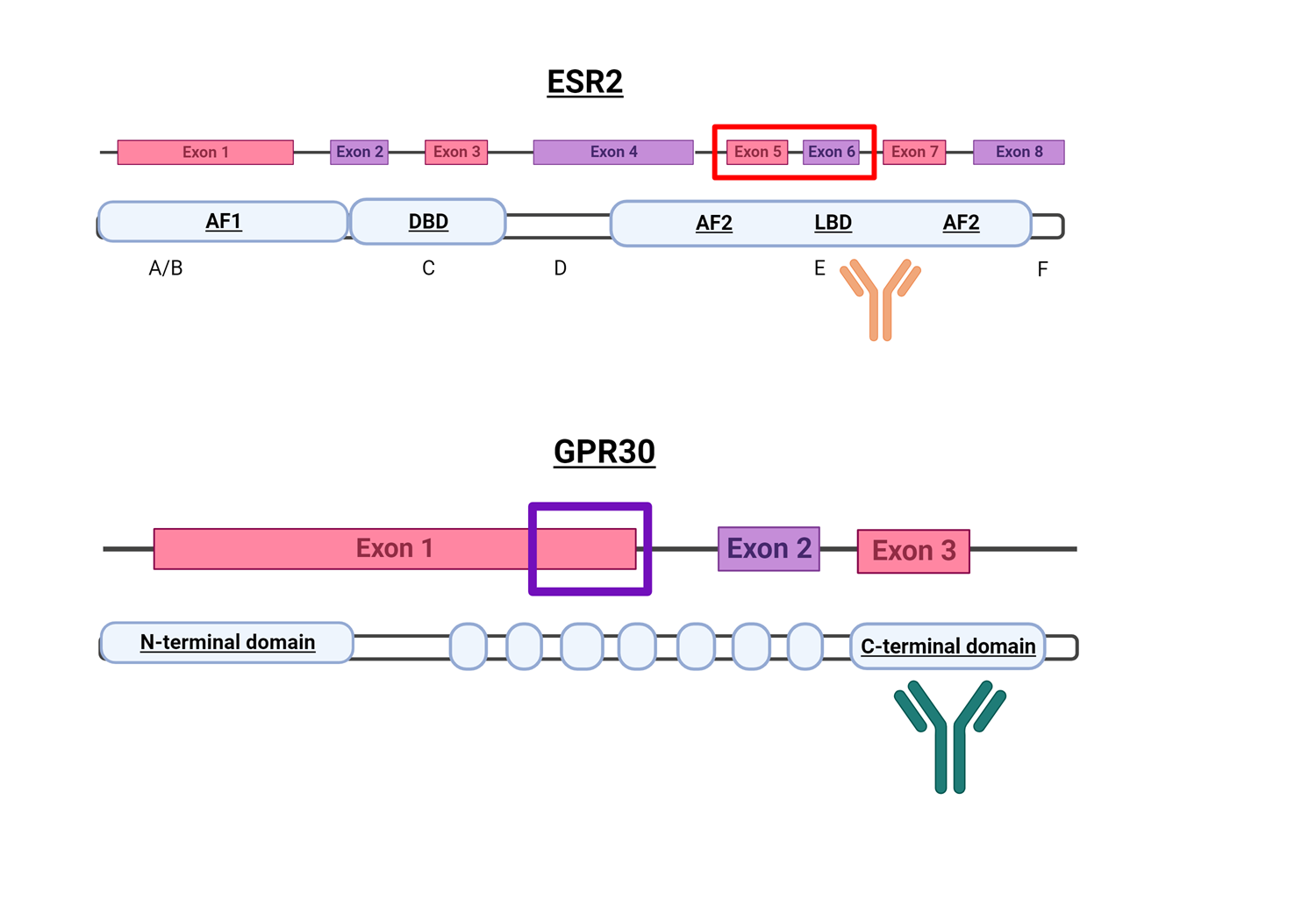
Supplemental Figure 2

**Supplemental Figure 2: Schematic of Estrogen Receptor beta and GPR30 primer and antibody targets.**


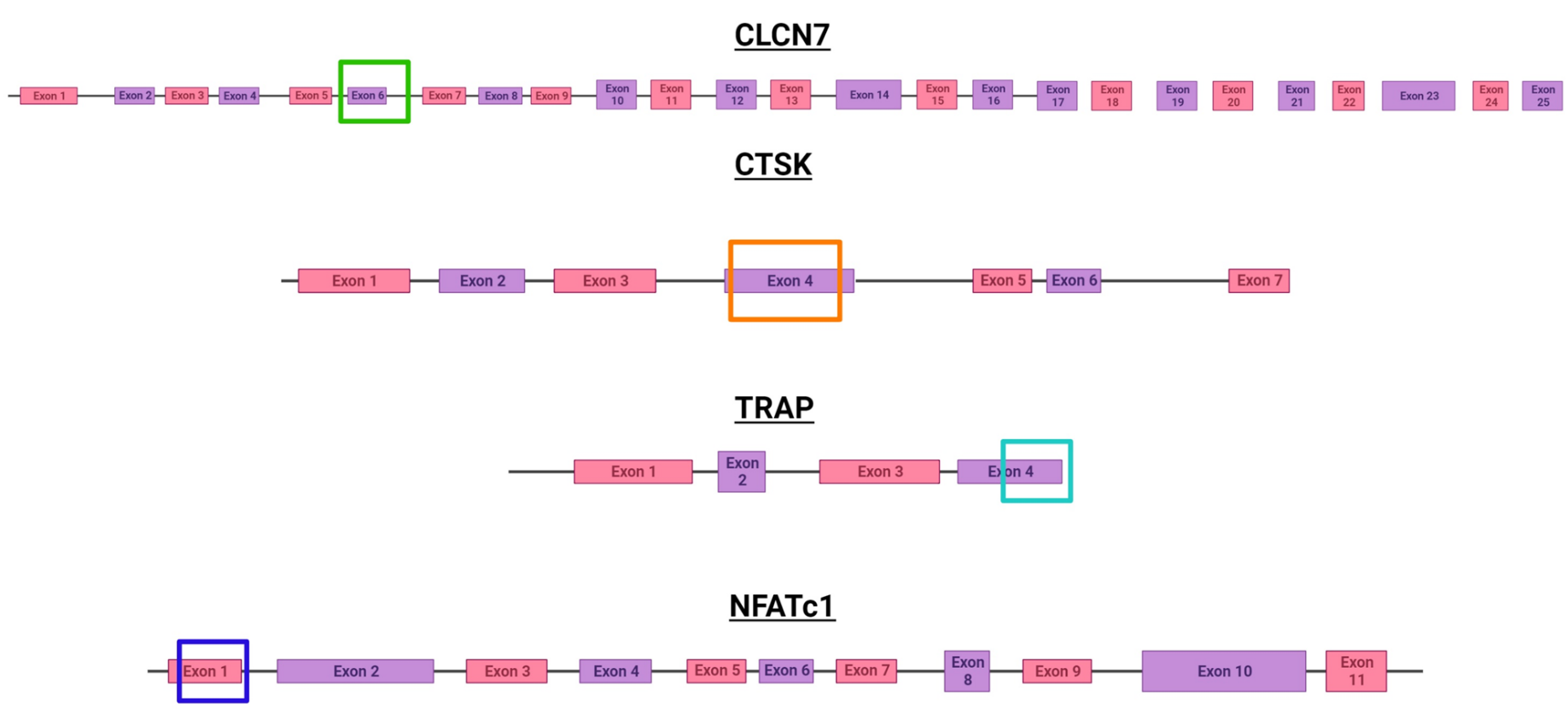
Supplemental Figure 3

**Supplemental Figure 3: Schematic of osteoclastogenic marker primer genes and targets.**


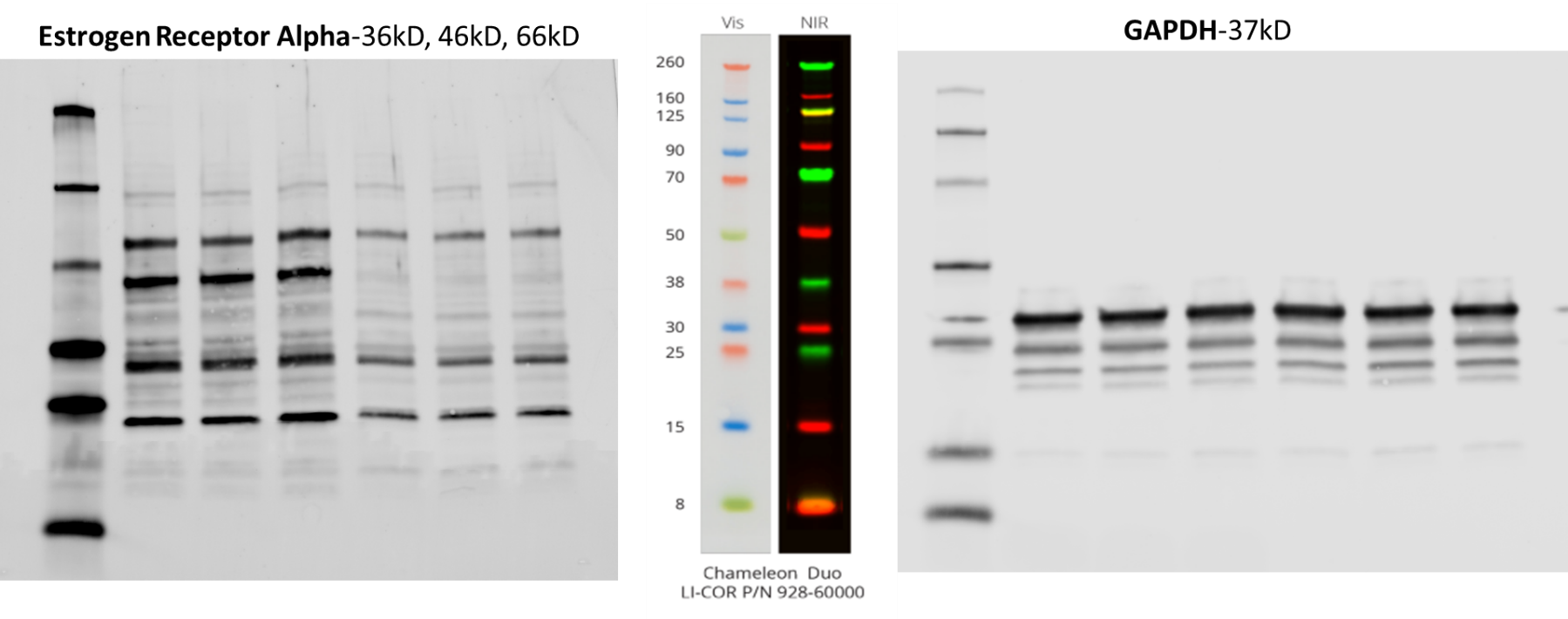
Supplemental Figure 4

MDA-MB-231

MCF7

MDA-MB-231

MCF7

**Supplemental Figure 4: Original western blots of estrogen receptor alpha protein levels in MCF7 and MDA-MB-231 cells.**

**
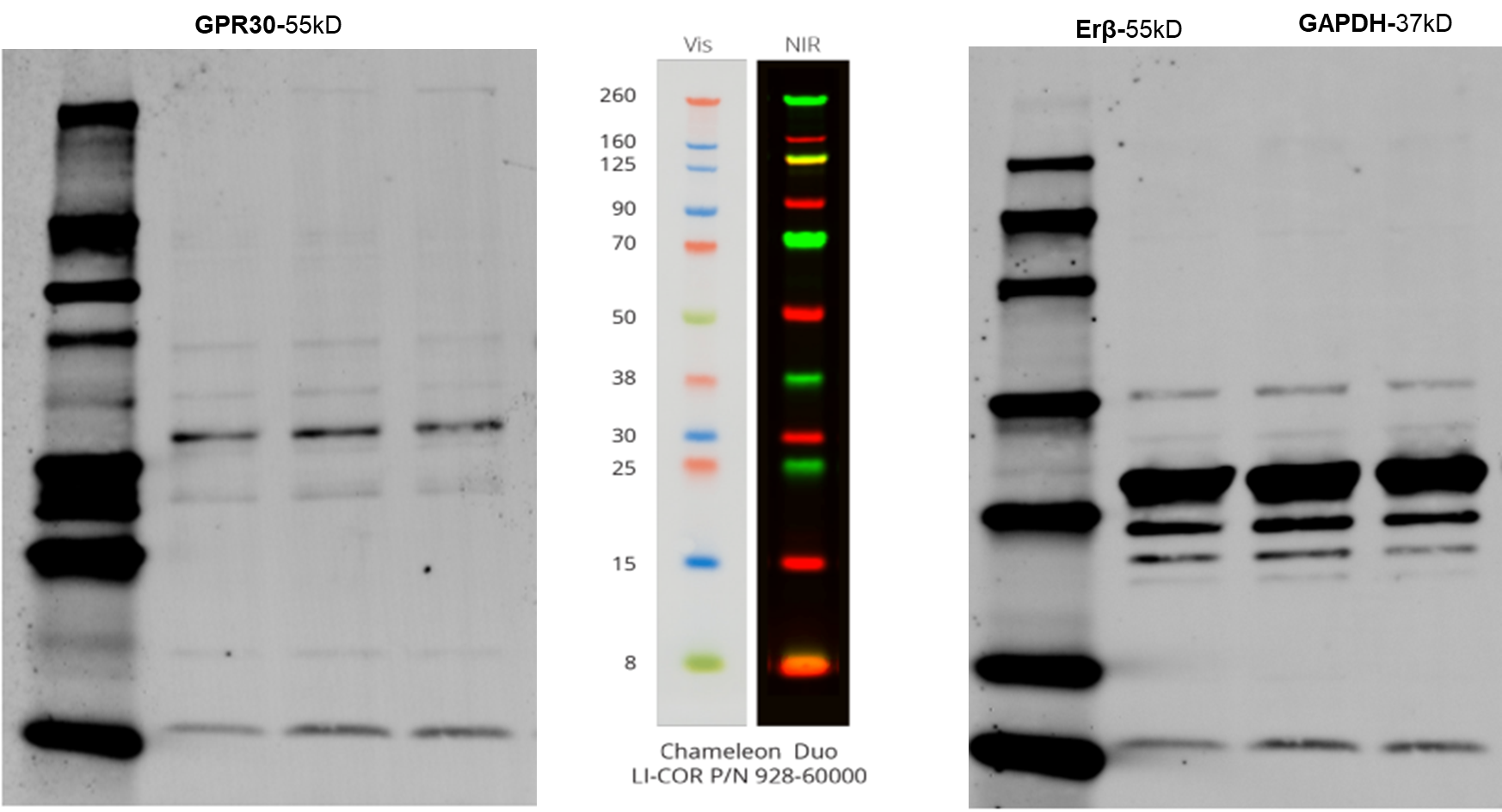
**Supplemental Figure 5

**Supplemental Figure 5: Original western blots of estrogen receptor beta and GPR30 protein levels in MDA-MB-231 cells.**

**
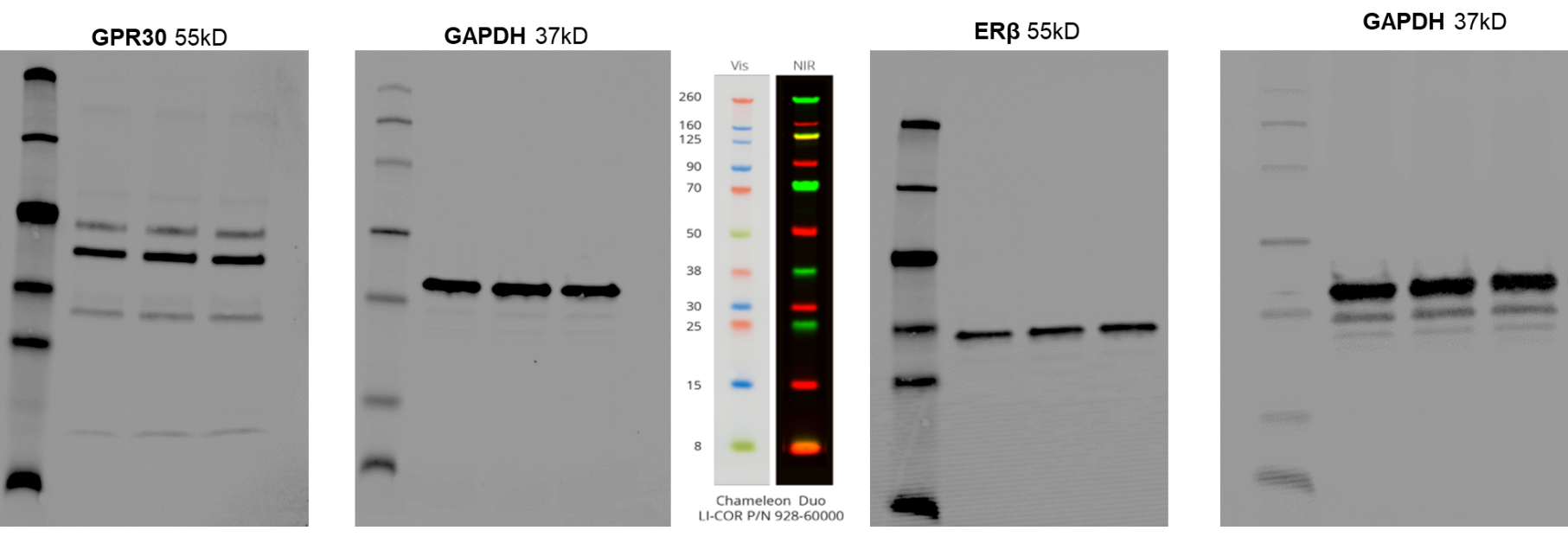
**Supplemental Figure 6

**Supplemental Figure 6: Original western blots of estrogen receptor beta and GPR30 protein levels in MCF7 cells.**

Supplemental Figure 7


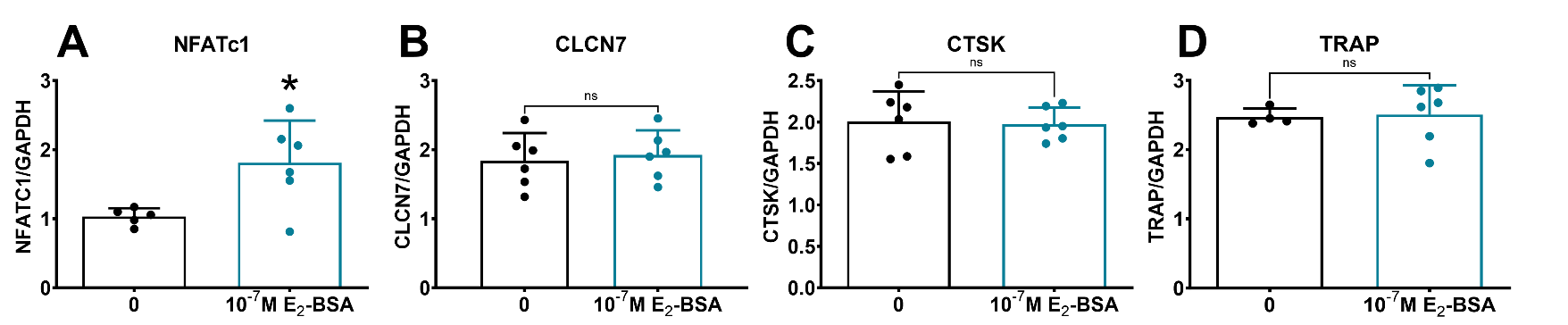


**Supplemental Figure 7: Effect of estrogen treated cancer cell conditioned media on osteoclast activation.** RAW264.7 cells were treated for 24 hours with conditioned media from MDA-MB-231 cells treated with or without E_2_-BSA for 9 minutes. Gene expression of NFATc1 (A), CLCN7 (B), CTSK (C), and TRAP (D) was measured. Groups labeled with ‘$’ are statistically significant compared to control with p values ≤0.05 considered significant by Student’s t-test.
